# Anesthetic Management Using Epidural Anesthesia and Continuous Intravenous Remifentanil in a Cesarean Section With Tethered Cord Syndrome: A Case Report

**DOI:** 10.7759/cureus.105370

**Published:** 2026-03-17

**Authors:** Kenichi Takechi, Aki Kawano, Tetsuya Takasaki, Erina Masaoka

**Affiliations:** 1 Department of Anesthesia, Matsuyama Red Cross Hospital, Matsuyama, JPN

**Keywords:** cesarean section, continuous intravenous remifentanil, epidural anesthesia, spina bifida, tethered cord syndrome

## Abstract

Spinal anesthesia is contraindicated in parturients with tethered cord syndrome undergoing cesarean sections. The epidural approach is an attractive anesthetic alternative. However, sacral complications can compromise their efficacy.

A 33-year-old parturient with a history of spina bifida and tethered cord syndrome was admitted for a cesarean section. Since imaging studies showed no abnormalities in the epidural space at the lumbar level, the anesthesiologist proposed epidural anesthesia. Continuous intravenous remifentanil provided additional analgesia. During uterine incision closure, the parturient complained of deep visceral pain. Consequently, the intravenous remifentanil dose was increased. The perioperative course for the parturient and neonate was uneventful.

For our parturient with tethered cord syndrome, the combination of epidural anesthesia and continuous intravenous remifentanil provided stable anesthesia during the cesarean section without apparent adverse effects to the parturient and neonate.

## Introduction

Tethered cord syndrome is a nervous system disorder caused by tissue that attaches to the spinal cord, limiting its movement. Its incidence is estimated at 0.25 per 1,000 births. Types include tight filum terminale, lipomeningomyelocele, split cord malformations, occult forms, dermal sinus tracts, and dermoids. Tethered cord syndrome is often associated with spina bifida. A parturient with tethered cord syndrome presents both obstetrical and anesthesia challenges.

Spinal anesthesia is the gold standard for cesarean sections [1]. However, it is difficult to administer to a parturient with tethered cord syndrome [2,3]. Traditionally, spinal anesthesia has been avoided for patients with tethered cord syndrome due to concerns of worsening neurologic disability. However, neuraxial anesthesia has several advantages over general anesthesia in high-risk cesarean sections [4,5].

Epidural anesthesia is an attractive option for cesarean sections where spinal anesthesia is contraindicated. However, sacral complications may compromise the efficacy of epidural anesthesia in the sacral region, which could complicate cesarean sections performed under epidural anesthesia.

This manuscript adheres to the applicable EQUATOR guidelines [6].

## Case presentation

**Figure 1:**
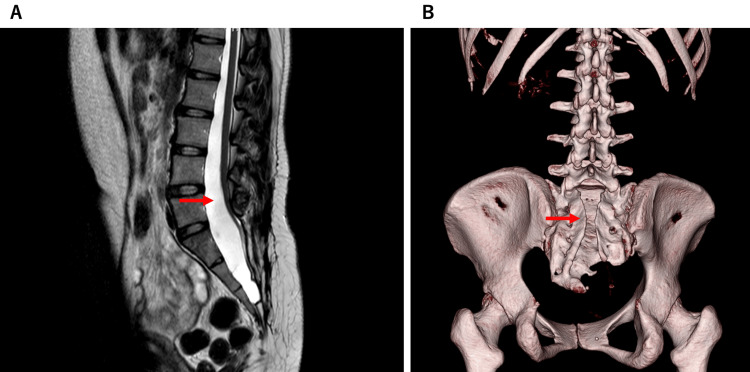
Lumbar and sacral images A. MRI shows a tethered spinal cord. The red arrow points to the low conus medullaris at the L5 level. B. Reconstructed CT images reveal spina bifida in the sacral region.

A 33-year-old parturient (height: 158.3 cm; weight: 68.7 kg) was admitted at 37 weeks of gestation. She had a history of spina bifida, tethered cord syndrome, and self-catheterization of the neurogenic bladder. She also had a history of laparoscopic sacrocolpopexy for uterine prolapse. A cesarean section was planned, and a conference was held between the obstetrician and anesthesiologist. The obstetrician explained that due to prior sacrocolpopexy, delivery might take longer than a normal cesarean section. Therefore, the effects of general anesthesia on the neonate were considered. As the MRI and CT images showed no abnormalities in the epidural space at the lumbar level (Figure 1), the anesthesiologist proposed the use of epidural anesthesia and continuous remifentanil for analgesia.

**Figure 2:**
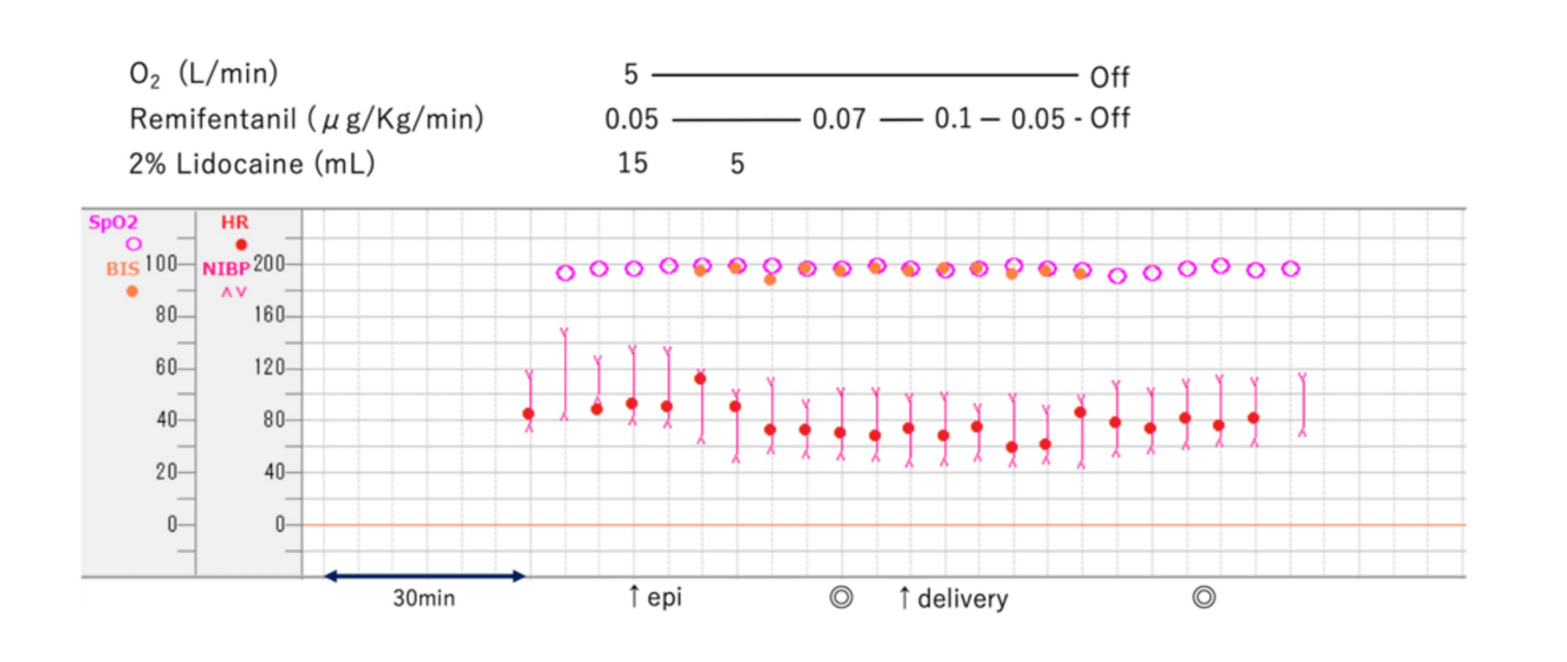
Anesthesia record SpO2, saturation of percutaneous oxygen, (%); BIS, bispectral index; HR, heart rate, (beat per minute); NIBP, non-invasive blood pressure, (mmHg); epi, epidural anesthesia; ◎ start and end of surgery

In the operating room, standard monitors were attached, and the parturient was placed in the right lateral decubitus position. A Tuohy needle was inserted at L2/3. The epidural space was confirmed using the loss-of-resistance technique with saline. After verifying the absence of blood and cerebrospinal fluid reflux, 15 mL of 2% lidocaine was administered via the Tuohy needle. Subsequently, an epidural catheter was placed. Fifteen minutes later, loss of cold sensation from T5 to L2 was confirmed. An additional 5 mL of 2% lidocaine was administered via the epidural catheter, and the loss of cold sensation at T4 was also confirmed at the start of surgery. Remifentanil was administered at a rate of 0.05 μg/kg/min after the placement of the epidural catheter and increased to 0.07 μg/kg/min at the start of surgery (Figure 2). The sedative side effects of remifentanil were monitored using electroencephalography (Bispectral Index; Medtronic-Covidien, Dublin, Ireland). We decided to administer remifentanil to ensure that the BIS score did not fall below 80. Oxygen was administered at a rate of 5 L/min, and exhaled carbon dioxide was monitored. The parturient did not experience any pain until delivery. The neonate was delivered nine minutes after the start of surgery. The neonatal Apgar score was four at one minute and nine at five minutes. During uterine incision closure, the parturient complained of dull pain. Consequently, remifentanil was increased to 0.1 μg/kg/min. Increasing the remifentanil dose alleviated deep visceral pain but caused mild drowsiness. There was no change in the parturient’s respiratory rate. The maternal-neonate first meeting and kangaroo care occurred without any problems. Remifentanil was tapered and discontinued after complete abdominal fascial closure. For postoperative pain management, 0.2% ropivacaine was administered epidurally at 3 mL/h until the first postoperative day. The postoperative course was uneventful. During the post-anesthesia rounds, the parturient did not recall any significant pain during the surgery.

## Discussion

This case report described a parturient with tethered cord syndrome who underwent cesarean section using epidural anesthesia combined with continuous remifentanil infusion.

Current guidelines recommend regional anesthesia, typically via spinal or epidural anesthesia, for cesarean sections [1]. There have been reports on safe spinal anesthesia for cesarean sections involving the spina bifida [7,8]. However, spinal anesthesia for tethered cord syndrome carries the risk of neurological complications [2,3]. A meta-analysis comparing general and neuraxial anesthesia for cesarean sections reported no clinically significant difference in neonatal outcomes [9]. Therefore, there is little justification for selecting spinal anesthesia for parturients at risk of spinal injury. Our parturient had a history of laparoscopic sacrocolpopexy, and the cesarean section delivery took longer. According to recent reports, neuraxial anesthesia yields better neonatal outcomes than general anesthesia during high-risk cesarean sections [4]. There were no anatomical abnormalities in the lumbar epidural space, and epidural anesthesia was deemed feasible. 

Epidural anesthesia acts segmentally. Its effect may be insufficient in cases where the diffusion of anesthetic agents into the sacral epidural region is difficult, such as in those with spina bifida. Parasympathetic nerve fibers from the pelvic visceral nerves enter the sacral region (S2-S4), which can cause deep visceral pain during cesarean sections [10]. Previous studies have shown that 50% of the parturients undergoing cesarean section under epidural anesthesia experience visceral pain [11]. For our parturient with spina bifida, the effectiveness of epidural anesthesia in the sacral region was anticipated to be incomplete.

Remifentanil crosses the placenta but is rapidly metabolized and/or redistributed, without adverse effects on the neonate [12]. A study has reported that remifentanil can be safely administered during vaginal delivery [13]. For cesarean sections, the combination of epidural anesthesia and continuous intravenous administration of low-dose remifentanil can significantly improve the labor experience, with no apparent adverse effects on the parturient or neonate [14]. Therefore, we used continuous remifentanil infusion and epidural anesthesia in this case. Increasing the infusion dose effectively alleviated deep visceral pain during surgery. However, its use in parturients remains controversial in terms of sedation and respiratory depression. Importantly, careful monitoring of respiration and consciousness is essential when continuous remifentanil infusion is used during cesarean sections [15].

## Conclusions

In conclusion, for a parturient with tethered cord syndrome undergoing cesarean section, the combination of epidural anesthesia and continuous intravenous remifentanil provided stable anesthesia, without apparent adverse effects on the parturient or neonate.

For caesarean sections involving parturients with a history of tethered cord syndrome, the combination of epidural anesthesia and continuous intravenous remifentanil is an attractive option. However, further data from additional cases are needed to assess safety and efficacy.
